# Hepcidin Regulation by BMP Signaling in Macrophages Is Lipopolysaccharide Dependent

**DOI:** 10.1371/journal.pone.0044622

**Published:** 2012-09-13

**Authors:** Xinggang Wu, Lai-Ming Yung, Wai-Hang Cheng, Paul B. Yu, Jodie L. Babitt, Herbert Y. Lin, Yin Xia

**Affiliations:** 1 School of Biomedical Sciences, The Chinese University of Hong Kong, Hong Kong, China; 2 Shenzhen Research Institute, The Chinese University of Hong Kong, Shenzhen, China; 3 Jinan University-The Chinese University of Hong Kong Key Laboratory for Regenerative Medicine, Ministry of Education, Guangzhou, China; 4 Brigham and Women's Hospital, Harvard Medical School, Boston, Massachusetts, United States of America; 5 Program in Anemia Signaling Research, Division of Nephrology, Program in Membrane Biology, Center for Systems Biology, Department of Medicine, Massachusetts General Hospital, Harvard Medical School, Boston, Massachusetts, United States of America; The Chinese University of Hong Kong, Hong Kong

## Abstract

Hepcidin is an antimicrobial peptide, which also negatively regulates iron in circulation by controlling iron absorption from dietary sources and iron release from macrophages. Hepcidin is synthesized mainly in the liver, where hepcidin is regulated by iron loading, inflammation and hypoxia. Recently, we have demonstrated that bone morphogenetic protein (BMP)-hemojuvelin (HJV)-SMAD signaling is central for hepcidin regulation in hepatocytes. Hepcidin is also expressed by macrophages. Studies have shown that hepcidin expression by macrophages increases following bacterial infection, and that hepcidin decreases iron release from macrophages in an autocrine and/or paracrine manner. Although previous studies have shown that lipopolysaccharide (LPS) can induce hepcidin expression in macrophages, whether hepcidin is also regulated by BMPs in macrophages is still unknown. Therefore, we examined the effects of BMP signaling on hepcidin expression in RAW 264.7 and J774 macrophage cell lines, and in primary peritoneal macrophages. We found that BMP4 or BMP6 alone did not have any effect on hepcidin expression in macrophages although they stimulated Smad1/5/8 phosphorylation and Id1 expression. In the presence of LPS, however, BMP4 and BMP6 were able to stimulate hepcidin expression in macrophages, and this stimulation was abolished by the NF-κB inhibitor Ro1069920. These results suggest that hepcidin expression is regulated differently in macrophages than in hepatocytes, and that BMPs regulate hepcidin expression in macrophages in a LPS-NF-κB dependent manner.

## Introduction

Hepcidin, a small cationic peptide, was first identified based on its antimicrobial and antifungal properties [1]; [2];[3]. Hepcidin provides a first line of defense at mucosal barriers, although it is not as potent as many other antimicrobial peptides [1]; [2];[3]. Recent studies demonstrated that hepcidin also acts as a major hormone to regulate iron homeostasis. Hepcidin negatively regulates iron in circulation by inhibiting iron absorption from the duodenum, iron recyling from the monocyte/macrophage system, and iron mobilization from hepatic stores. Hepcidin blocks iron efflux by binding to the sole iron exporter ferroportin and inducing its internalization and degradation. Hepcidin expression is dramatically increased during infection and inflammation. This leads to a marked decrease in serum iron, thus depriving microbes of iron and decreasing their rate of growth [3].

Hepcidin is mainly synthesized by the liver. The expression of hepcidin in hepatocytes increases in response to infection/inflammation and elevated systemic iron. In isolated primary hepatocytes, hepcidin expression is stimulated by IL-6, IL-1 and lipopolysaccharide (LPS), but not by TNF-α [4]; [5]; [6]. Recently, we and others discovered that bone morphogenetic protein (BMP) signaling pathway is critically involved in regulating hepcidin expression in the liver [7]; [8]; [9]; [10]; [11]. BMPs signal through type II and type I serine threonine kinase receptors, which phosphorylate intracellular receptor-activated Smad proteins (Smad1/5/8). Phosphorylated Smad1/5/8 then bind to the common mediator Smad4, and the Smad complex translocates to the nucleus to regulate transcription of target genes such as Id1 [12]; [13]. BMP2, 4, 5, 6, and 9 have been shown to induce hepcidin expression in isolated murine primary hepatocytes or in hepatoma cell lines [14]; [9]; [10]. Administration of BMP2 or BMP6 increases hepcidin expression and decreases serum iron levels in mice [9]; [11]. Conversely, inhibition of BMP signaling through genetic deletion of the ligand BMP6 [11]; [15], the BMP type I receptor ALK3 [16], the BMP co-receptor hemojuvelin (Hjv) [7], or Smad4 [8], or through administration of BMP ligand antagonists HJV.Fc [9] or ALK3-Fc, or the BMP type I receptor inhibitor LDN-193189 [16], results in low hepcidin expression in the liver in mice. Thus, BMP signaling is an important regulatory pathway for hepcidin expression in hepatocytes.

Hepcidin is also expressed by myeloid cells including monocytes, macrophages and neutrophils [17]; [18]; [19]; [20]; [21]; [22]; [23]. In contrast to hepatocytes, hepcidin expression in macrophages is not induced by iron loading in mice [17]. Interestingly, expression of hepcidin in myeloid cells is increased under stimulation of bacterial pathogens and LPS, and this stimulation is dependent on TLR4 and NF-κB activities [18]; [22]; [23]. The autocrine and paracrine effects of hepcidin secreted by monocytic lineages is to decrease iron efflux and promote iron retention in these cells [19]; [21]. In fact, hepcidin-mediated iron loading in macrophages and monocytes appears to increase their inflammatory potential both *in vitro* and *in vivo* [24], consistent with its other functions in promoting host defense. Considering 90–95% of the serum iron comes from recycling of iron from damaged erythrocytes by macrophages, the autocrine/paracrine action of hepcidin in macrophages may be an important mechanism in innate immune defense by which the host reduces the availability of iron from pathogens, while simultaneously augmenting the activity of innate immune effector cells.

Based on the pivotal role of BMP signaling in hepcidin regulation in hepatocytes, we investigated whether or not BMP signaling regulates hepcidin expression in macrophages. Surprisingly, we found that macrophages did not increase hepcidin expression in response to BMP stimulation, unless BMP signaling was accompanied by co-stimulation with LPS.

## Materials and Methods

### Chemical and biochemical reagents

BMP4 and BMP6 were purchased from R & D Systems (Minneapolis, MN). Lipopolysaccharides (LPS) from Escherichia coli 0127:B8 was purchased from Sigma-Aldrich (Saint Louis, MO). Phospho-Smad1/5/8, Smad1, phospho-NF-κB p65 and NF-κB p65 antibodies were purchased from Cell Signaling Technology (Beverly, MA). β-actin antibodies were purchased from Sigma-Aldrich. NF-κB inhibitor Ro1069920 was purchased from Santa Cruz Biotechnology, Inc (Santa Cruz, CA).

### Cell Culture

RAW264.7 and J774 macrophage cell lines (ATCC) were cultured in Dulbecco's modified Eagle's medium (DMEM) supplemented with 10% heat-inactivated fetal bovine serum (Invitrogen), 2 mM L-glutamine, and antibiotics. Cells were starved with serum-free DMEM supplemented with 0.1% bovine serum albumin before they were incubated with BMP4, BMP6, LPS or a combination of BMP ligands and LPS as indicated. Cells were lysed for real-time PCR quantification of mRNA transcripts or for Western blotting analyses for phosporylated Smad1/5/8 and phosphorylated NF-κB p65 levels.

Mature resident peritoneal macrophage were harvested and cultured as previously described [25]. Briefly, C57BJ/L mice were sacrificed by cervical dislocation. Under sterile conditions, midline incision was made and abdominal skin was retraced gently with forceps. 10 ml sterile PBS was injected slowly into the abdominal cavity. Peritoneal fluids were withdrawn slowly. Approximately 6–8 ml of fluid were recovered from each mouse. Lavage fluids from several mice were pooled and cells were collected by centrifugation at 400 g for 10 min. Cell were counted and cultured in DMEM supplement with 10% FCS at a density of 6×10^5^ cells/ml. Primary macrophage were allowed to attach overnight and fed with fresh medium to culture for another day. Cells were starved in DMEM supplemented with 1% FCS overnight and then incubated with BMP4 or BMP6 and/or LPS in FCS-free DMEM supplemented with 0.1% BSA as indicated. Cells were harvested and phospho-Smad1/5/8 levels or hepcidin mRNA levels were measured. All the procedures were performed in accordance with Animal Experimentation Ethics Approval by Brigham and Women's Hospital Animal Experimentation Ethics Committee.

### Transfection

To examine the effect of HJV overexpression on hepcidin expression in RAW264.7 macrophages, cells were transfected with Flag-HJV cDNA in 12-well plates using Lipofectamine 2000 (Invitrogen). Approximately 24 h after transfection, the media was replaced with serum-free DMEM medium supplemented with 0.1% BSA. 16 h later, the cells were harvested for measurement of hepcidin mRNA expression.

### Measurement of Gene Expression

Real-time quantification of mRNA transcripts was performed using an AB 7600 real-time system (Applied Biosystems). First-strand cDNA was amplified with previously described mouse hepcidin and Id1 primers (11), and detected using SYBR® Green PCR Master Mix (Applied Biosystems) according to the manufacturer's instructions. In parallel, Rpl19 (ribosomal protein-like 19) transcripts were amplified and detected in a similar manner to serve as an internal control. Standard curves were generated from accurately determined dilutions of plasmid cDNAs or purified PCR fragments as templates. Results are expressed as a ratio of the gene of interest to Rpl19.

### Western blotting

RAW264.7 cells or peritoneal primary macrophages were lysed in TBS (50 mM Tris-HCl, 150 mM NaCl, and 1% Triton X-100 [pH 7.4]) containing protease inhibitor mixture (Pierce) and phosphatase inhibitor mixture (Pierce) for 30 min on ice. After centrifugation for 10 min at 4°C, the supernatant was assayed for protein concentration by colorimetric assay (BCA kit; Pierce). A total of 20–40 µg of protein was separated by SDS-PAGE and transferred to polyvinylidene difluoride membranes. Membranes were probed with anti–phospho-Smad1/5/8 (1∶1000) or anti–phospho-NF-κB p65 (1∶1000). Membranes were stripped in 0.2 M glycine (pH 2.5) and 0.5% Tween 20 for 10 min, and reprobed with anti-Smad1 (1∶1000), anti-NF-κB p65 (1∶1000) or anti-β-actin (1∶10,000) antibodies.

### Data analysis

Results from real time PCR analyses are expressed as the means ± S.D. of three replicates. Differences were assessed by Student's *t* test with *P*<0.05 used to indicate significance.

## Results

### BMP signaling does not stimulate hepcidin expression in macrophages

**Figure 1:**
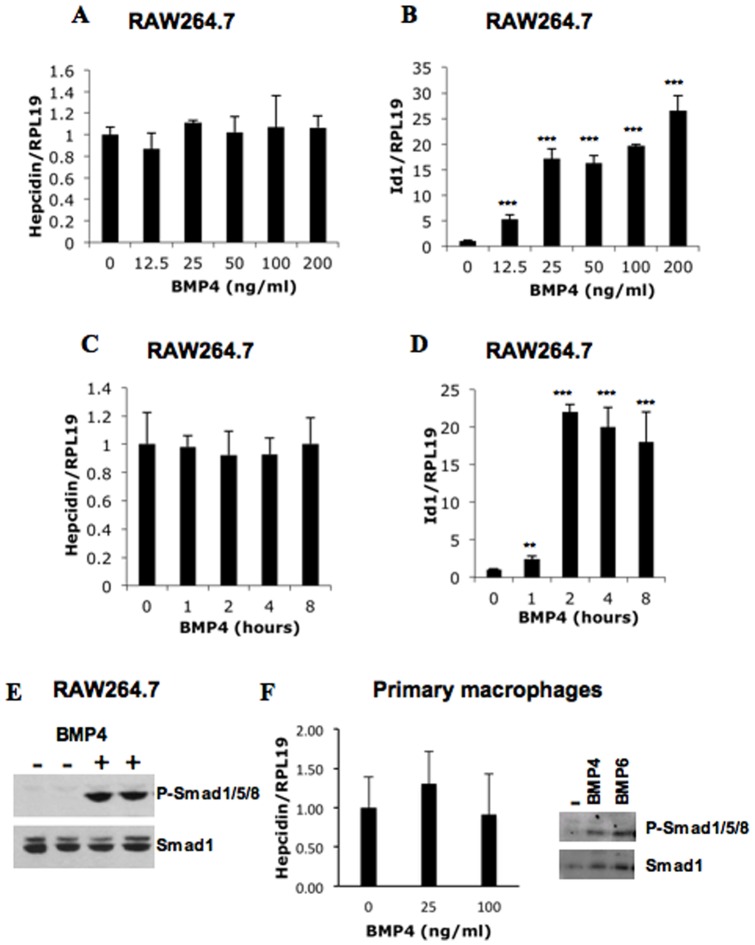
Effects of BMP4 on hepcidin expression in macrophages. (A, B, C and D) Effects of BMP4 on hepcidin and Id-1 expression in RAW264.7 macrophages. Cells were incubated overnight with increasing amounts of BMP4 (A & B) or with 50 ng/ml of BMP4 for increasing periods of time (C &D) in serum free DMEM supplemented with 0.1% BSA. Cells were collected for measurements of hepcidin (A & C), Id1 (B & D) and RPL19 mRNA levels. (E) Effects of BMP4 on phosphorylation levels of Smad1/5/8 in RAW264.7 macrophages. Cells were incubated with BMP4 for 1 h before cells were lysed for Western blotting for phospho-Smad1/5/8 and Smad1 levels. (F) Left panel: Effects of BMP4 on hepcidin expression in primary mouse peritoneal macrophages. Cells were incubated for 8 h with increasing amounts of BMP4 before cells were collected for quantification of hepcidin and RPL19 mRNA levels. Right panel: Effects of BMP4 and BMP6 on phosphorylation levels of Smad1/5/8 in primary mouse peritoneal macrophages. Cells were incubated with BMP4 or BMP6 for 1 h before cells were lysed for Western blotting for phospho-Smad1/5/8 and Smad1 levels. **, *P*<0.01; ***, *P*<0.001.

It is well documented that BMPs stimulate hepcidin expression in hepatocytes. To examine whether or not BMP signaling plays a role in hepcidin expression in macrophages, we incubated RAW264.7 macrophages with BMP4. Surprisingly, hepcidin mRNA levels were not stimulated by BMP4 with concentrations of up to 200 ng/ml (Fig. 1A), while expression of Id1 mRNA, a well-known target gene of the BMP pathway, was increased by BMP4 in a dose-dependent manner (Fig. 1B). A time course of 0 to 8 h for BMP4 treatment (50 ng/ml) also demonstrated no change in hepcidin expression (Fig. 1C), despite an increase in Id1 expression and phosphorylated Smad1/5/8 levels 1 h after BMP4 treatment (Fig. 1D &1E). Similar results were seen with J774 macrophages (data not shown). In addition, BMP4 did not alter hepcidin expression in primary peritoneal macrophages isolated from mice, while it induced phosphorylated Smad1/5/8 levels (the left and right panels, Fig. 1F).

**Figure 2:**
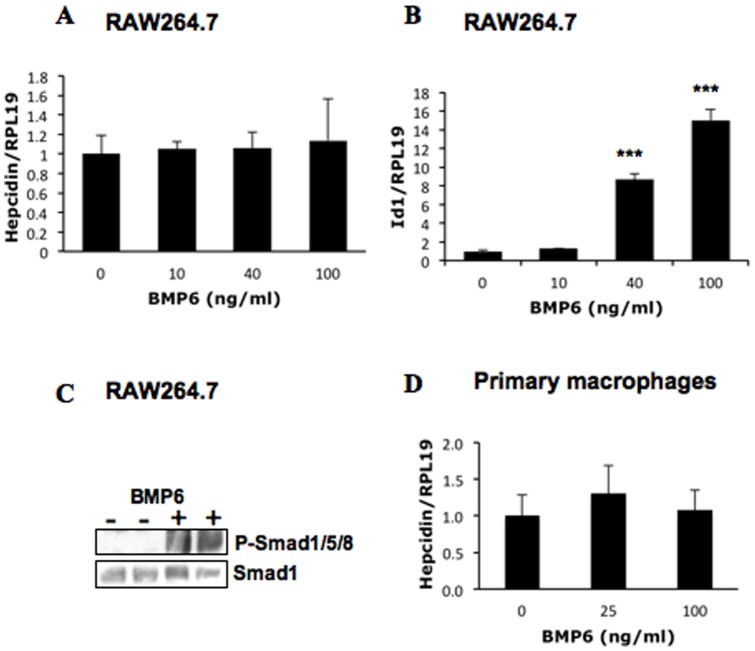
Effects of BMP6 on hepcidin expression in RAW264.7 macrophages. (A and B) Cells were incubated for 8 h with increasing amounts of BMP6 in serum free DMEM supplemented with 0.1% BSA. Cells were collected for real time RT-PCR analyses to qualify hepcidin (A), Id1 (B) and RPL19 mRNA levels. (C) Effects of BMP6 on phosphorylation levels of Smad1/5/8 in RAW264.7 macrophages. Cells were incubated with BMP6 for 1 h before cells were lysed for Western blotting for phospho-Smad1/5/8 and Smad1 levels. (D) Effects of BMP6 on hepcidin expression in primary mouse peritoneal macrophages. Cells were incubated for 8 h with increasing amounts of BMP6 before cells were collected for quantification of hepcidin and RPL19 mRNA levels. ***, *P*<0.001.

BMP6 appears to be a key endogenous BMP ligand for hepcidin expression in the liver [11]; [15]. Therefore, we also examined whether BMP6 could regulate hepcidin expression in macrophages. BMP6 failed to stimulate hepcidin expression in RAW264.7 macrophages (Fig. 2A), while it dramatically increased Id1 expression (Fig. 2B) and stimulated phosphorylated Smad1/5/8 levels (Fig. 2C). Similarly, BMP6 did not have any effect on hepcidin expression in primary peritoneal macrophages (Fig. 2D), while it stimulated Smad1/5/8 phosphorylation (the right panel, Fig. 1F). In addition, BMP6 did not affect hepcidin expression in J774 macrophages either (data not shown).

**Figure 3:**
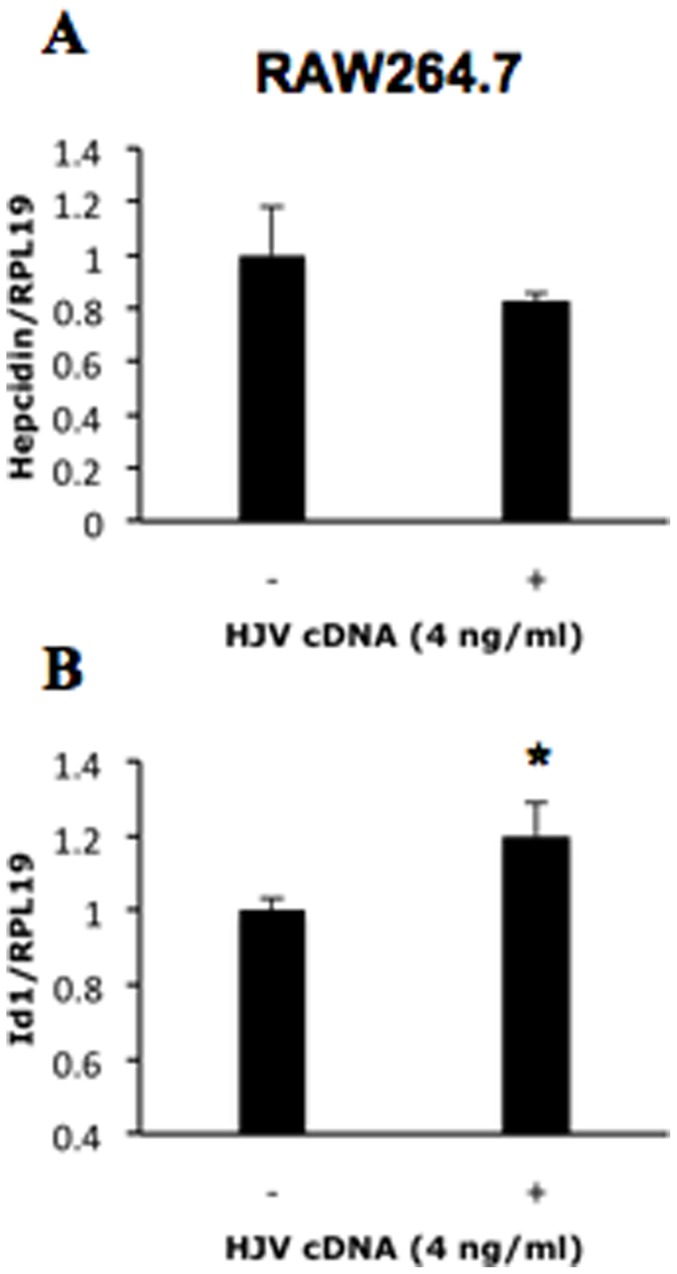
Effects of HJV on hepcidin expression in RAW264.7 macrophages. Cells were tranfected with HJV cDNA (4 ng/ml). 48 hours after transfection, the cells were collected for real time RT-PCR analyses to qualify hepcidin (A) and Id1 (B). *, *P*<0.05.

HJV is a co-receptor for BMP signaling that increases BMP signaling and hepcidin expression in hepatocytes. Our previous study showed that expression of endogenous HJV in RAW264.7 cells is low [26]. Overexpression of HJV did not alter hepcidin expression in RAW264.7 cells (Fig. 3A), although it was sufficient to potentiate Id1 expression (Fig. 3B), suggesting that the lack of a response to BMP stimulation was not attributable to decreased expression of this co-receptor in RAW264.7 cells.

These results suggest that, unlike hepatocytes, macrophages do not respond to BMP stimulation with an increase in hepcidin expression. This unresponsiveness cannot be attributed to defects in BMP signaling in macrophages because Smad1/5/8 phosphorylation and Id1 expression were induced by treatment with BMP4 or BMP6, and Id1 expression was induced by overexpression of HJV.

### BMP4 and BMP6 stimulate hepcidin expression in macrophages in the presence of LPS

**Figure 4:**
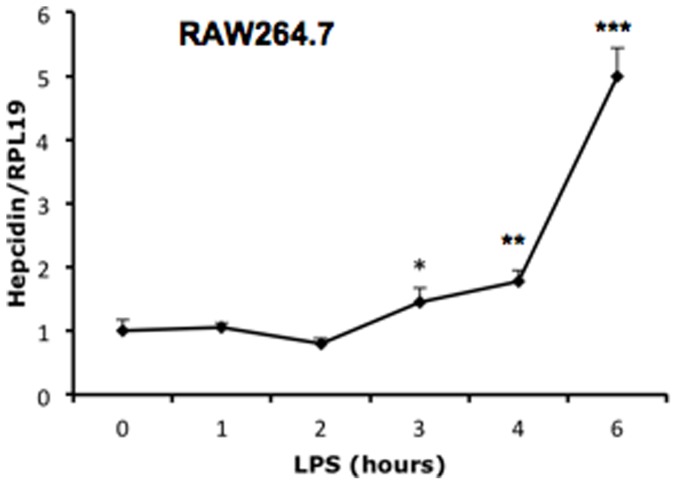
Effects of LPS on hepcidin expression in RAW264.7 macrophages. Cells were incubated with LPS (10 ng/ml) in serum free DMEM supplemented with 0.1% BSA, and were collected at the indicated times for real time RT-PCR analyses to qualify hepcidin and RPL19 mRNA levels. *, *P*<0.05; **, *P*<0.01; ***, *P*<0.001.

**Figure 5:**
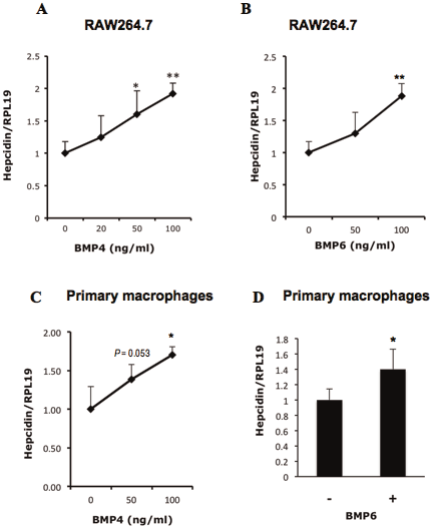
Effects of BMP4 on hepcidin expression in macrophages in the presence of LPS. (A and B) RAW264.7 cells were incubated with increasing amounts of BMP4 (A) or BMP6 (B) in the presence of LPS (10 ng/ml) for 8 h. Cells were collected for real time RT-PCR analyses to qualify hepcidin and RPL19 mRNA levels. (C and D) Primary mouse peritoneal macrophages were incubated with BMP4 (C) or BMP6 (D) in the presence of LPS (10 ng/ml) for 8 h. Cells were collected for real time RT-PCR analyses to qualify hepcidin and RPL19 mRNA levels. (B) *, *P*<0.05; **, *P*<0.01.

Hepcidin in macrophages is stimulated by bacterial pathogens and LPS [18]. Consistent with these observations, LPS significantly increased hepcidin expression in RAW264.7 cells in a time-dependent manner (Fig. 4). To examine whether BMP can influence hepcidin expression in macrophages activated by LPS, we incubated RAW264.7 cells (Fig. 5A & B) or primary peritoneal macrophages (Fig. 5C & D) with varying concentrations of BMP4 or BMP6 in the presence of LPS. In the presence of LPS co-stimulation, BMP4 and BMP6 were able to stimulate hepcidin expression in both the RAW264.7 macrophage cell line and primary peritoneal macrophages. These results suggest that additional intracellular signals are required for BMP regulation of hepcidin expression in macrophages.

### The stimulation of hepcidin expression by BMP signaling in the presence of LPS is dependent on the NF-κB pathway

**Figure 6:**
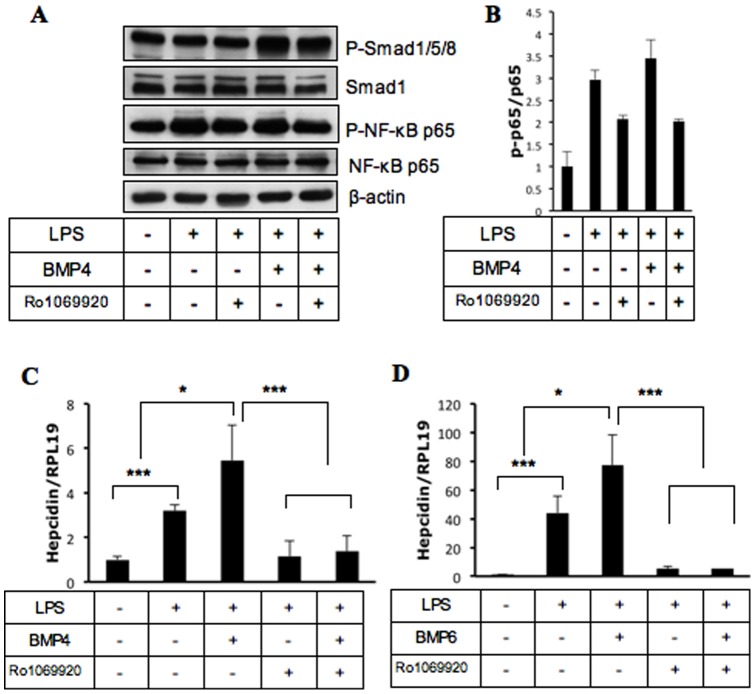
Effects of the NF-κB inhibitor Ro1069920 on hepcidin expression induced by LPS and BMP4 in RAW264.7 macrophages. (A and B) Cells were incubated for 8 h with LPS alone, or LPS in combination with BMP4 in the presence or absence of Ro1069920 (5 µM) in serum free DMEM supplemented with 0.1% BSA. Cells were collected for Western blotting analyses using the indicated antibodies (A), and densitometry analysis from two replicates was performed (B). (C and D) Cells were incubated for 8 h with LPS alone, or LPS in combination with BMP4 (C) or BMP6 (D) in the presence or absence of Ro1069920 (5 µM) in serum free DMEM supplemented with 0.1% BSA. Cells were collected for real time RT-PCR analyses to qualify hepcidin and RPL19 mRNA levels. *, *P*<0.05; ***, *P*<0.001.

A previous study showed that LPS-induced hepcidin expression is mediated by the NF-κB signaling pathway in human leukocytes [23]. To examine whether the NF-κB is also involved in hepcidin expression induced by LPS and BMP4 in macrophages, we used the NF-κB inhibitor Ro1069920 [27]. As shown in Fig. 6A and 6B, this inhibitor reduced phosphorylation levels of NF-κB p65 induced by LPS, but did not affect phosphorylation levels of Smad1/5/8 induced by BMP4 in RAW264.7 macrophages. While LPS stimulated hepcidin expression more than 3-fold above basal levels (Fig. 6C, bar 2), the addition of BMP4 further increased hepcidin mRNA levels (Fig. 6C, bar 3). Incubation with Ro1069920 reduced hepcidin levels stimulated by LPS alone (Fig. 6C, bar 4) or by BMP4 in combination with LPS (Fig. 6C, bar 5) to basal levels. Similarly, BMP6 in combination of LPS increased hepcidin expression over LPS alone (compare bars 2 and 3, Fig. 6D). In the presence of Ro1069920, hepcidin levels were dramatically inhibited and they were no longer different between cells treated with LPS alone and with BMP6 plus LPS (compare bars 4 and 5, Fig. 6D). Taken together, these results suggest that the potentiation of LPS-induced hepcidin expression by BMP signaling in macrophages is dependent on NF-κB co-signaling.

## Discussion

Hepcidin enhances the host immune defense by its antimicrobial and antifungal activities, and by its negative regulation of serum iron levels. Hepcidin is mainly produced by the liver, but it is also expressed in myeloid cells including macrophages and monocytes. Infections stimulate hepcidin expression in both hepatocytes and myeloid cells, leading to an increase in hepcidin concentrations in circulation and in urine. Although the contribution of myeloid cells to the circulating hepcidin relative to that of hepatocytes during infections is difficult to determine, emerging evidence suggests that hepcidin produced from macrophages and monocytes under the stimulation of bacterial pathogens or LPS inhibits the growth of bacteria while inducing iron retention in these cells. Depletion of iron from monocytic lineage impairs LPS-induced expression of inflammatory cytokines including interferon β, whereas iron retention appears to potentiate these cytokine responses [24]. Therefore, the paracrine/autocrine actions of hepcidin may play an important role in the innate immunity mediated by macrophages and monocytes.

A number of differences in the regulatory mechanisms of hepcidin expression have been identified for myeloid cells and hepatocytes. For example, hepcidin expression in macrophages in the spleen is not regulated by serum iron levels, while hepatocytes increase hepcidin expression in response to iron loading. In leukocytes [23] and lymphocytes [28], TNF-α is potent in inducing hepcidin expression. In hepatocytes, however, IL-6 and IL-1 stimulate hepcidin expression while TNF-α does not have such an effect [4]; [6]; [29]; [30]; [31]. In the present study, we have identified another difference between hepatocytes and macrophages, i.e. unlike hepatocytes, macrophages did not increase hepcidin expression in response to BMP signaling under basal conditions. In our experiments, the exposure of RAW264.7 or J774 macrophages to two distinct BMP ligands (BMP4 and BMP6) at varying doses and duration, the transfection of RAW264.7 with HJV, a BMP co-receptor and potent hepcidin inducer in hepatocytes, or the stimulation of primary peritoneal macrophages with BMP4 and BMP6 all failed to induce hepcidin expression.

The mechanism underlying the failure of BMP signaling to stimulate hepcidin expression in macrophages remains unknown. Phosphorylation of Smad1/5/8 and Id1 expression was stimulated by BMP4 or BMP6 in macrophages. This suggests that the BMP signaling pathway is not defective. Whether there are any transcription factors that are important for hepcidin transcription but are not present or not activated in macrophages, and whether there are any epigenetic differences in the hepcidin promoter between hepatocypes and macrophages remain to be addressed. A recent study showed that the binding motif of HNF4α, a liver specific transcription factor, in the hepcidin promoter is critical for the HJV and BMP response of hepcidin in hepatocytes [32]. However, we failed to rescue the BMP response of hepcidin expression by forced expression of HNF4α in RAW264.7 macrophages (data not shown).

STAT3 is a key transcription factor involved in regulation of hepcidin in hepatocytes in inflammatory conditions [30]; [31]. However, in neutrophils or leukocytes, inhibition of STAT3 did not have any effect on LPS-induced hepcidin expression, while inhibition of NF-κB activity abolished the stimulation of hepcidin expression by LPS. Therefore, hepcidin expression induced by LPS in neutrophils or leukocytes depends on the NF-κB pathway [23]. In fact, there is a NF-κB binding site in the proximal region of the mouse hepcidin promoter, and mutation of the NF-κB site completely abolished hepcidin promoter activity in RAW264.7 cells under the stimulation of *M. tuberculosis*, suggesting a critical role of NF-κB for hepcidin promoter activity [22]. In the present study, we found that, in the presence of LPS co-stimulation, BMP4 stimulated hepcidin expression in RAW264.7 and primary peritoneal macrophages. Interestingly, the potentiation of LPS-induced hepcidin by BMP4 or BMP6 was completely abrogated by inhibition of NF-κB activity in RAW264.7 macrophages. These results suggest that the action of LPS on hepcidin expression is also dependent on NF-κB activity in macrophages, and that NF-κB pathway plays a key role in determining the BMP response of hepcidin expression in macrophages. A previous study showed that inhibition of NF-κB signaling pathway completely blocked the induction of iNOS expression by BMP6 in RAW264.7 cells [33]. These results suggest that the crosstalk between the NF-κB pathway and the Smad pathway may be important for macrophage functions.

The mechanisms responsible for the effects of BMPs on hepcidin expression in the presence of LPS co-stimulation were not examined in the present study. Previous studies have shown that LPS induces SOCS3 expression, and SOCS3 inhibits hepcidin expression as a negative feedback. Studies have also shown that TGF-β1 inhibits SOCS3 expression [34]. It is tempting to speculate that BMP signaling may also inhibit SOCS3 expression, thus potentiating LPS action in stimulating hepcidin expression.

Augmented BMP signaling via the enhanced expression of BMP ligands, Smad effectors or their downstream activation, has been frequently observed to be a hallmark of infection, inflammation and local tissue injury [35]; [36]; [37]; [38]. Just as it is possible that the activation of pathogen associated molecular pattern (PAMP) signaling pathways via molecules such as LPS might adaptively recruit innate immune responses in synergy with tissue damage signals marked by BMP ligand signaling, it is also possible that the absence of BMP signals may limit inflammation when it is inappropriate. We speculate further that this novel mechanism of cooperative signaling between BMP and LPS in regulating hepcidin in monocytic lineages might be exploited therapeutically to enhance immunity or dampen inflammation via pharmacologic BMP signaling modulation.
